# Evaluation of HLA-G 14 bp Ins/Del and +3142G>C Polymorphism with Susceptibility and Early Disease Activity in Rheumatoid Arthritis

**DOI:** 10.1155/2016/4985745

**Published:** 2016-08-16

**Authors:** Mohammad Hashemi, Mahnaz Sandoughi, Seyed Amirhossein Fazeli, Gholamreza Bahari, Maryam Rezaei, Zahra Zakeri

**Affiliations:** ^1^Cellular and Molecular Research Center, Zahedan University of Medical Sciences, Zahedan 98167-43181, Iran; ^2^Department of Clinical Biochemistry, School of Medicine, Zahedan University of Medical Sciences, Zahedan 98167-43181, Iran; ^3^Department of Internal Medicine, School of Medicine, Zahedan University of Medical Sciences, Zahedan 98167-43181, Iran; ^4^Department of Internal Medicine, School of Medicine, Shahid Beheshti University of Medical Sciences, Tehran 19857-17443, Iran

## Abstract

*Purpose/Background*. Mounting evidence designates that HLA-G plays a role in the regulation of inflammatory processes and autoimmune diseases. There are controversial reports concerning the impact of* HLA-G* gene polymorphism on rheumatoid arthritis (RA). This study was aimed at examining the impact of 14 bp ins/del and +3142G>C polymorphism with susceptibility and early disease activity in RA patients in a sample of the Iranian population.* Methods*. This case-control study was done on 194 patients with RA and 158 healthy subjects. The* HLA-G* rs1063320 (+3142G>C) and rs66554220 (14 bp ins/del) variants were genotype by polymerase chain reaction-restriction fragment length polymorphism (PCR-RFP) and PCR method, respectively.* Results*. The* HLA-G* +3142G>C polymorphism significantly decreased the risk of RA in codominant (OR = 0.61, 95% CI = 0.38–0.97, *p* = 0.038, GC versus GG; OR = 0.36, 95% CI = 0.14–0.92, *p* = 0.034, CC versus GG), dominant (OR = 0.56, 95% CI = 0.36–0.87, *p* = 0.011, GC + CC versus GG), and allele (OR = 0.58, 95% CI = 0.41–0.84, *p* = 0.004, C versus G) inheritance models tested. Our finding did not support an association between* HLA-G* 14 bp ins/del variant and risk/protection of RA. In addition, no significant association was found between the polymorphism and early disease activity.* Conclusion*. In summary, our results showed that* HLA-G* +3142G>C gene polymorphism significantly decreased the risk of RA in a sample of the Iranian population.

## 1. Introduction

Rheumatoid arthritis (RA) is the most common autoimmune disease of unknown etiology affecting approximately 0.5–1% of the human population worldwide [1, 2]. The disease is 2-3 times more common in females than in males. It has been proposed that both genetic and environmental factors are involved in the expression and complications of the disease [3–8]. Genetic factors are assumed to contribute to up to 60% of the risk of developing RA [2].

Human leucocyte antigen-G (HLA-G), a nonclassical major HLA class Ib molecule, may suppress functions of natural killer (NK) cells, CD4+, CD8+ lymphocytes, and dendritic cell [9–11]. HLA-G protein potentially exists as seven isoforms including four membrane-bound (HLA-G1, -G2, -G3, and -G4) as well as three secreted soluble (HLA-G5, -G6, and -G7) proteins [12].

HLA-G gene, which is located on chromosome 6 (6p21.31), contains a 14 bp insertion (ins)/deletion (del) and a +3142G>C (rs1063320) polymorphism in 3′-untranslated region (3′UTR) of HLA-G. HLA-G expression rate and plasma level are influenced by polymorphism in the promoter region as well as 3′-untranslated region (UTR) variants [13–15].

A 14 bp ins/del polymorphism in exon 8 in the 3′UTR of* HLA-G* was found to be associated with the stability and splicing patterns of* HLA-G* mRNA isoforms. The homozygous deletion of 14 bp confers a more stable mRNA as compared to the homozygous insertion genotype [13, 14, 16]. Low levels of membrane bound and sHLA-G levels are associated with the ins allele [13].

+3142G>C polymorphism influences the affinity of HLA-G mRNA targeted by different microRNAs as demonstrated by an in silico study [17]. +3142G allele has a binding site with higher affinity for miR-148a, miR-148b, and miR-152 downregulating the expression of HLA-G [15, 18].

The common polymorphism of the* HLA-G* seems to affect its level of expression and may have an impact on disease susceptibility in autoimmune disorders. It has been reported that plasma soluble HLA-G (sHLA-G) levels were lower in RA patients than in controls [19].

Several studies investigated the impact of common polymorphism of HLA-G (+3142G>C and 14 bp ins/del) on RA risk in various population, but the findings have been controversial [20–24]. Therefore, the present study was aimed at examining whether rs1063320 (+3142G>C) and rs66554220 (14 bp ins/del) polymorphism in the* HLA-G* gene were associated with susceptibility to RA in a sample of Iranian population.

## 2. Material and Methods

### 2.1. Patients

A total of 352 subjects including 194 patients with RA fulfilling the 2010 American College of Rheumatology/European League Against Rheumatism for RA [25] and 158 unrelated healthy subjects were enrolled in the study. The cases were selected from RA patients admitted to the Rheumatology Clinic of university-affiliated hospital (Ali-Ebne-Abitaleb Hospital, Zahedan, Iran). The control group consisted of 158 whose age and sex matched healthy individuals with no clinical symptoms or family histories of RA, and they were unrelated to RA patients, had no known autoimmune diseases, and were from the same geographical origin as the patients with RA (Zahedan, Iran). The project was approved by local ethics committee of Zahedan University of Medical Sciences and informed consent was obtained from all participants. Genomic DNA was extracted from peripheral blood samples using salting out method as described previously [26].

Among all the participant patients, 30 early RA subjects who were symptomatic for ≤1 year enrolled for subsequent follow-up study. All the patients were on standard therapeutic regimen. The disease activity was determined by disease activity score 28 (DAS-28) at the beginning and the end of the follow-up study (at least 18 months) by the same specialist rheumatologist. At the end of the study, the patients were stratified into remitting (DAS-28 < 2.6) and nonremitting (DAS-28 ≥ 2.6) patients.

**Figure 1:**
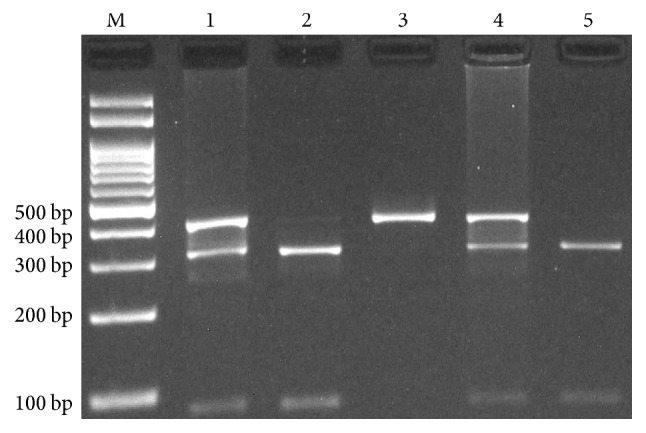
Photograph of the PCR products of HLA-G +3142G>C polymorphism by polymerase chain reaction-restriction fragment length polymorphism method (PCR-RFLP). G allele digested by BaeGI restriction enzyme and produced 316 bp and 90 bp while C allele undigested 406 bp. M: DNA Marker; Lanes 1 and 4: GC; Lanes 2 and 5: GG; Lane 3: CC.

Genotyping of HLA-G rs1063320 (+3142G>C) variant was performed by PCR-RFLP methods. The set of forward and reverse primers were 5′-CATGCTGAACTGCATTCCTTCC-3′ and 5′-CTGGTGGGACAAGGTTCTACTG-3′ [27]. Amplification was done with an initial denaturation step at 95°C for 5 min, followed by 30 cycles of 30 s at 95°C, 30 s at 65°C, and 30 s at 72°C with a final step at 72°C for 10 min. 10 *μ*L of PCR products was digested with BaeGI restriction enzyme (Fermentas). G allele digested and produced 316 bp and 90 bp (digested), while C allele undigested and produced 406 bp (Figure 1).

**Figure 2:**
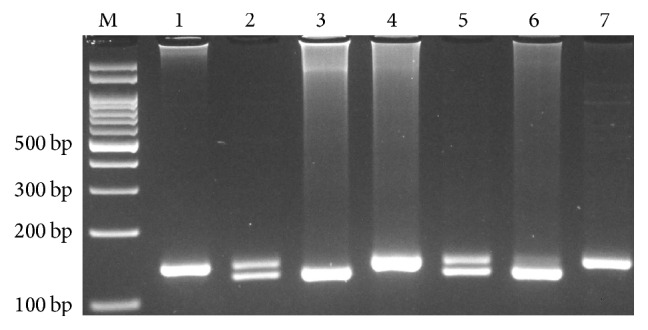
Photograph of the PCR products of* HLA-G* 14 bp ins/del polymorphism by polymerase chain reaction (PCR). Product sizes were 127 bp for del and 141 bp for ins allele. M: DNA Marker; Lanes 1, 4, and 7: ins/ins; Lanes 2 and 5: ins/del; Lanes 3 and 6: del/del.

Genotyping of HLA-G rs66554220 (14 bp ins/del) variant was done by polymerase chain reaction [28]. The forward and reverse primers were 5′-TCACCCCTCACTGTGACTGATA-3′ and 5′-GCACAAAGAGGAGTCAGGGTT-3′, respectively. In each 0.20 mL PCR reaction tube, 1 *μ*L of genomic DNA (~100 ng/mL), 1 *μ*L of each primer (10 *μ*M), 10 *μ*L of 2x Prime Taq Premix (Genet Bio, Korea), and 5 *μ*L ddH_2_O were added. The PCR cycling conditions were as follows: an initial denaturation step of 5 min at 95°C followed by 30 cycles of 30 s at 95°C, annealing at 56°C for 30 s, and extension at 72°C for 30 s, with final extension at 72°C for 5 min. The PCR products were separated by electrophoresis in 2% agarose gels and observed under ultraviolet light. Product sizes were 127 bp for del and 141 bp for ins allele (Figure 2).

### 2.2. Statistical Analysis

Statistical analysis of the data was done using statistical software package SPSS 20 software. Independent sample *t*-test for continuous data and *χ* ^2^ test for categorical data were used. The associations between genotypes of* HLA-G* gene and RA were assessed by computing the odds ratio (OR) and 95% confidence intervals (95% CI) from logistic regression analyses. Haplotype analysis was performed using SNPStats software (a web tool for the analysis of association studies). *p* value less than 0.05 was considered statistically significant. The Bonferroni correction was applied by multiplying *p* values by the number of SNPs analyzed.

## 3. Results

In this study, we recruited 194 RA patients (180 female and 14 male; mean age 45.3 ± 14.1 years) and 158 unrelated healthy subjects (140 female and 18 male; mean age: 46.1 ± 12.3 years). There was no significant difference between the groups concerning sex and age (*p* = 0.815 and *p* = 0.465, resp.).

The genotype and allele frequencies of* HLA-G* polymorphism in RA patients and in controls are shown in Table 1.* HLA-G* rs1063320 (+3142G>C) variant decreased the risk of RA in codominant (OR = 0.61, 95% CI = 0.38–0.97, *p* = 0.038, GC versus GG; OR = 0.36, 95% CI = 0.14–0.92, *p* = 0.034, CC versus GG) and dominant (OR = 0.56, 95% CI = 0.36–0.87, *p* = 0.011, GC + CC versus GG) tested inheritance models.* HLA-G* rs1063320 C allele significantly decreased the risk of RA (OR = 0.58, 95% CI = 0.41–0.84, *p* = 0.004) compared to G allele.

Overall, both chi-square comparison and logistic regression analysis (which was calculated in each model of inheritance) did not reveal an association between* HLA-G* rs66554220 polymorphism and RA risk (Table 1).

In the combined analysis of two* HLA-G* variants, subjects carrying deldel/GG genotypes had significantly higher risk of RA than 14 bp deldel/+3142GG (Table 2).

Haplotype analysis is shown in Table 3. Haplotype +3142G/14 bp del significantly increased the risk of RA (OR = 1.77, 95% CI = 1.14–2.75, *p* = 0.012), while +3142C/14 bp del decreased the risk of RA (OR = 0.52, 95% CI = 0.30–0.90, *p* = 0.019) compared to +3142G/14 bp ins.

Baseline demographic and clinical characteristics of total follow-up cohort and the remitting and nonremitting subgroups are shown in Table 4. We determined the association of HLA-G polymorphism with early disease activity. Our results revealed no significant association between* HLA-G* +3142G>C and HLA-G 14 bp ins/del variant and early disease activity (Table 5).

The genotype frequency of the HLA-G polymorphism was examined for Hardy-Weinberg equilibrium (HWE). +3142G>C polymorphism in cases and controls was in HWE (*χ* ^2^ = 0.50, *p* = 0.480 and *χ* ^2^ = 0.96, *p* = 0.328, resp.), while the 14 bp I/D variant in cases and controls was not in HWE (*χ* ^2^ = 13.94, *p* = 0.0002 and *χ* ^2^ = 8.38, *p* = 0.004, resp.).

## 4. Discussion

HLA-G is a nonclassical HLA class I molecule that can bind to immune cells and inhibit their function [29, 30]. It is involved in several immunoregulatory processes and may potentially be involved in the pathogenesis of RA. Genetic variants in coding and noncoding regions of the* HLA-G* may affect biological features of the molecule. Expression rate of* HLA-G* gene and plasma level are affected by variants in the promoter region as well as 3′UTR [12].

In the present study, we investigated the impact of* HLA-G* 14 bp ins/del and +3142G>C polymorphism on risk of RA in a sample of Iranian population. The findings of our study showed an association between* HLA-G* +3142G>C polymorphism and RA in our population. The GC as well as C allele decreased the risk of RA in our population. Regarding* HLA-G* 14 bp ins/del variant, we did not find any statistically significant difference in either genotype or allele distribution between patients and controls. The deldel/GG genotypes significantly increased the risk of RA compared to insins/GG. In addition, we did not find an association between* HLA-G* variants and disease activity. In contrast to our findings, Rizzo et al. [31] investigated 23 early rheumatoid arthritis (ERA) patients during a 12-month follow-up disease treatment period. They found that the frequency of 14 bp del allele was associated with disease remission. They concluded that HLA-G may be a candidate biomarker to evaluate early prognosis and disease activity in ERA patients.

A meta-analysis performed by Lee et al. [32] revealed no significant association between* HLA-G* 14 bp I/D and +3142G/C polymorphism and RA risk. Similar negative findings have been reported in Brazilian [24] and Indian population [22]. Although Veit et al. [23] have observed no differences in allelic and genotypic frequencies of the* HLA-G* 14 bp ins/del polymorphism between RA patients and controls, the 14 bp ins/del polymorphism was associated with juvenile idiopathic arthritis in Brazilian population. In another study, Veit et al. [33] reported that +3142GG genotype significantly increased the risk of RA (odds ratio (OR) = 1.45, 95% confidence interval (CI) = 1.075–1.95, *p* = 0.030). Kim et al. [20] investigated the impact of rs1736936 (-1202T/C) and rs2735022 (-586C/T) promoter polymorphism of* HLA*-*G* gene on RA in Korean population. They found no significant differences in distributions of genotypes and haplotypes between RA patients and control subjects.

Verbruggen et al. [19] found that the levels of sHLA-G in patients with RA were significantly lower than healthy controls. They suggested that patients with low sHLA-G levels were unable to suppress self-reactive cells leading to development of autoimmunity. The 3′-untranslated region (UTR) has a major role in HLA-G regulation [17, 34]. It has been proposed that polymorphism exerts a significant effect in the* HLA-G* function and may have an impact on the expression of sHLA-G [35–37]. The HLA-G expression is influenced by 14 bp ins/del as well as +3142G/C polymorphism in the 3′-untranslated region (3′UTR) of HLA-G gene and may have possible implications of clinical significance [37].

The discrepancy in findings among studies may be due to genetic and environmental differences between the different populations being investigated.

The limitation of our study is that we have no data regarding anti-CCP antibodies, RF antibody, HLA-DRB1 shared epitope, and smoking history. Consequently, we could not evaluate the association between* HLA-G* variants and these factors. However, we believe that our findings provide an important input into the debate concerning the clinical relevance of studied variants. There is no clear explanation for deviation from HWE in our population. The possible reason is that the HLA-G gene is under balancing selection [34].

In summary, we found a significant association between* HLA-G* +3142G>C variant and susceptibility to RA in a sample of Iranian population. Further association studies with large sample size and different ethnicities are required to verify our findings.

## Figures and Tables

**Table 1:** Association of HLA-G polymorphisms and the risk of RA.

HLA-G polymorphisms	Case *n* (%)	Control *n* (%)	OR (95% CI)	*p*	*p* ^c^
*14-bp ins/del (rs66554220)*					
Codominant					
Ins/ins	36 (18.6)	34 (21.5)	1.00	—	
Ins/del	123 (63.4)	97 (61.4)	1.20 (0.70–2.05)	0.582	1.00
Del/del	35 (18.0)	27 (17.1)	1.22 (0.62–2.43)	0.603	1.00

Dominant					
Ins/ins	36 (18.6)	34 (21.5)	1.00		
Ins/del + del/del	158 (81.4)	124 (78.5)	1.20 (0.71–2.03)	0.505	1.00

Recessive					
Ins/ins + ins/del	159 (82.0)	131 (82.9)	1.00		
Del/del	35 (18.0)	27 (17.1)	1.07 (0.61–1.86)	0.888	1.00

Allele					
Ins	195 (50.3)	165 (52.2)	1.00	—	
Del	193 (49.7)	151 (47.8)	1.08 (0.80–1.46)	0.649	1.00

*+3142G>C (rs1063320) *					
Codominant					
GG	135 (69.6)	89 (56.3)	1.00	—	
GC	52 (26.8)	56 (35.4)	0.61 (0.38–0.97)	0.038	0.076
CC	7 (3.6)	13 (8.2)	0.36 (0.14–0.92)	0.034	0.068

Dominant					
GG	135 (69.6)	89 (56.3)	1.00		
GC + CC	59 (30.4)	69 (43.7)	0.56 (0.36–0.87)	0.011	0.022

Recessive					
GG + GC	187 (96.4)	145 (91.8)	1.00		
CC	7 (3.6)	13 (8.2)	0.42 (0.16–1.07)	0.068	1.00

Allele					
G	322 (83.0)	234 (74.0)	1.00	—	
C	66 (17.0)	82 (26.0)	0.58 (0.41–0.84)	0.004	0.005

*p* ^c^: Bonferroni-corrected *p*.

**Table 2:** Interaction of 14 bp ins/del and +3142G>C polymorphism of *HLA-G* gene on rheumatoid arthritis (RA) risk.

14 bp ins/del	+3142G>C	RA cases *n* (%)	Controls *n* (%)	OR (95% CI)	*p*	*p* ^c^
Ins/ins	GG	27 (13.9)	27 (17.1)	1.00	—	—
Ins/del	GG	84 (43.3)	56 (35.4)	1.50 (0.79–2.82)	0.257	1.000
Del/del	GG	24 (12.4)	6 (3.8)	4.00 (1.41–11.34)	0.010	0.039
Ins/del	GC	34 (17.5)	35 (22.2)	0.97 (0.48–1.98)	0.890	1.000
Del/del	GC	10 (5.2)	14 (8.9)	0.71 (0.27–1.89)	0.624	0.992
Ins/ins	GC	8 (4.1)	7 (4.4)	1.14 (0.36–3.60)	0.922	1.000
Del/del	CC	1 (0.5)	7 (4.4)	0.14 (0.02–1.24)	0.063	0.240
Ins/del	CC	5 (2.6)	6 (3.8)	0.83 (0.23–3.06)	0.927	1.000
Ins/ins	CC	1 (0.5)	0 (0.0)	—	—	—

*p* ^c^: Bonferroni-corrected *p*.

**Table 3:** Haplotype association of *HLA-G* +3142G>C and 14 bp ins/del variants with rheumatoid arthritis (RA) risk.

+3142G>C	14 bp ins/del	RA cases (frequency)	Controls (frequency)	OR (95% CI)	*p*
G	Ins	0.4250	0.4652	1.00	—
G	Del	0.4049	0.2753	1.77 (1.14–2.75)	0.012
C	Del	0.0925	0.2026	0.52 (0.30–0.90)	0.019
C	Ins	0.0776	0.0569	1.74 (0.75–4.05)	0.200

**Table 4:** Baseline demographic and clinical characteristics of total follow-up cohort and the remitting and nonremitting subgroups.

Parameters	Total patients (*n* = 30)	Remitting patients (*n* = 15)	Nonremitting patients (*n* = 15)	*p*
Age (mean ± SD)	45.56 ± 16.99	46.26 ± 17.22	44.86 ± 17.34	NS^*∗*^

Gender (%)				
Male	2 (6.7)	2 (13.3)	0 (0.0)	NS
Female	28 (93.3)	13 (86.6)	15 (100.0)	

*BMI (Kg/m* ^2^) (mean ± SD)	25.18 ± 5.24	24.87 ± 3.34	25.52 ± 6.84	NS

Cigarette (pack/years; mean ± SD)	0.33 ± 1.82	0.00 ± 0.00	0.66 ± 2.58	

Hookah (%)	4 (13.3)	1 (6.6)	3 (20)	NS

Education				NS
Illiterate (%)	12 (40.0)	6 (40.0)	6 (40.0)	
Less than diploma (%)	5 (16.7)	1 (6.6)	4 (26.7)	
Diploma (%)	8 (26.6)	4 (26.7)	4 (26.7)	
Higher education (%)	5 (16.7)	4 (26.7)	1 (6.6)	

Length of symptom prior to study (months; mean ± SD)	8.20 ± 4.22	8.20 ± 4.63	8.20 ± 3.94	NS

Positive rheumatoid factor (%)	26 (86.7)	12 (80)	14 (93.3)	NS

Comorbidity				NS
No comorbidity (%)	17 (56.6)	7 (80.0)	10 (66.6)	
Type 2 diabetes mellitus (%)	3 (10.0)	1 (6.6)	2 (13.3)	
Hypertension (%)	6 (20.0)	5 (33.3)	1 (6.6)	
Dyslipidemia (%)	6 (20)	2 (13.3)	4 (26.6)	
Other factors (%)	5 (16.6)	4 (26.6)	1 (6.6)	

^*∗*^Nonsignificant.

**Table 5:** Association of *HLA-G* polymorphism in remitting and nonremitting RA patients.

Genotypes	Remitting patients *n* (%)	Nonremitting patients *n* (%)	OR (95% CI)	*p*
*14-bp ins/del*				
Genotype				
Ins/ins	1 (50.0)	1 (50.0)	1.00	—
Ins/del	10 (43.5)	13 (56.5)	0.76 (0.04–13.88)	0.897
Del/del	4 (80.0)	1 (20.0)	4.00 (0.12–137.10)	0.912

Allele				
Ins	12 (40.0)	15 (50.0)	1.00	—
Del	18 (60.0)	15 (50.0)	1.50 (0.54–4.17)	0.602

*+3142G>C*				
Genotype				
GG	11 (45.8)	13 (54.2)	1.00	—
CG	4 (66.7)	2 (33.3)	2.36 (0.36–15.46)	0.651
CC	0 (0.0)	0 (0.0)	—	—

Allele				
G	26 (63.4)	28 (65.1)	1.00	—
C	15 (36.6)	15 (34.9)	0.90 (0.38–2.14)	0.826
